# Saracatinib Inhibits Middle East Respiratory Syndrome-Coronavirus Replication In Vitro

**DOI:** 10.3390/v10060283

**Published:** 2018-05-24

**Authors:** Jin Soo Shin, Eunhye Jung, Meehyein Kim, Ralph S. Baric, Yun Young Go

**Affiliations:** 1 Virus Research Group, Korea Research Institute of Chemical Technology, Daejeon 34114, Korea; jsshin@krict.re.kr (J.S.S.); e-hyeya@hanmail.net (E.J.); mkim@krict.re.kr (M.K.); 2 Department of Biological Sciences, Korea Advanced Institute of Science and Technology, Daejeon 34141, Korea; 3 Department of Medicinal Chemistry and Pharmacology, University of Science and Technology, Daejeon 34114, Korea; 4 Department of Epidemiology, University of North Carolina at Chapel Hill, Chapel Hill, NC 27599, USA; rbaric@email.unc.edu

**Keywords:** Middle East Respiratory Syndrome, MERS-CoV, Src-family kinase inhibitor, saracatinib, gemcitabine

## Abstract

The Middle East respiratory syndrome-coronavirus (MERS-CoV), first identified in Saudi Arabia, is an emerging zoonotic pathogen that causes severe acute respiratory illness in humans with a high fatality rate. Since its emergence, MERS-CoV continues to spread to countries outside of the Arabian Peninsula and gives rise to sporadic human infections following the entry of infected individuals to other countries, which can precipitate outbreaks similar to the one that occurred in South Korea in 2015. Current therapeutics against MERS-CoV infection have primarily been adapted from previous drugs used for the treatment of severe acute respiratory syndrome. In search of new potential drug candidates, we screened a library composed of 2334 clinically approved drugs and pharmacologically active compounds. The drug saracatinib, a potent inhibitor of Src-family of tyrosine kinases (SFK), was identified as an inhibitor of MERS-CoV replication in vitro. Our results suggest that saracatinib potently inhibits MERS-CoV at the early stages of the viral life cycle in Huh-7 cells, possibly through the suppression of SFK signaling pathways. Furthermore, saracatinib exhibited a synergistic effect with gemcitabine, an anticancer drug with antiviral activity against several RNA viruses. These data indicate that saracatinib alone or in combination with gemcitabine can provide a new therapeutic option for the treatment of MERS-CoV infection.

## 1. Introduction

The emergence and re-emergence of infectious diseases represent a significant threat to global public health and socioeconomic stability as witnessed by numerous outbreaks over the past few decades [1]. The Middle East respiratory syndrome-coronavirus (MERS-CoV), first identified in Saudi Arabia in 2012, was the second zoonotic introduction of a highly pathogenic coronavirus into the human population within a decade [2]. Since its emergence, MERS-CoV continues to spread to countries outside of the Arabian Peninsula and has caused epidemics with high fatality rates [1,3].

The MERS-CoV is a member of the genus *Betacoronavirus* in the family Coronaviridae [4,5]. Primary transmission of MERS-CoV is suspected to occur through close contact between humans and infected animal reservoirs such as dromedary camels [6,7]. Most infections have occurred in Middle Eastern countries that are associated with human-to-human spread, typically starting in healthcare settings that result in sporadic outbreaks [8]. The clinical features of MERS-CoV infection in humans range from asymptomatic to severe lower respiratory tract infections with the potential development of acute respiratory distress syndrome, septic shock, and multiorgan failure resulting in death [9,10]. Due to high morbidity and mortality rates, therapeutic options of MERS-CoV were immediately adapted from previous reports of severe acute respiratory syndrome (SARS) therapies including the use of broad-spectrum antibiotics, corticosteroids, interferons, ribavirin, lopinavir-ritonavir, and/or mycophenolate mofetil, but none of them were effective in randomized controlled trials [3,11]. Therefore, the rapid discovery of effective prophylactic or therapeutic measures to prevent or treat MERS is urgently needed.

Repurposing clinically validated products is a valuable approach for drug discovery, which has the potential to greatly reduce the time and costs associated with the development and licensure of de novo therapeutics [12]. Prompt response and availability of a therapeutic option are especially critical for controlling the spread of highly infectious pathogens such as MERS-CoV. Along these lines, several Food and Drug Administration (FDA)-approved compounds have been reported to inhibit MERS-CoV replication in cell culture since its emergence [13,14,15,16,17].

In search of additional MERS-CoV inhibitors, we conducted a screening of 2334 approved drugs and biologically active molecules using a cytopathic-effect (CPE)-based, high-throughput screening (HTS) assay. From the results, we identified saracatinib, a potent inhibitor of the Src-family of tyrosine kinases (SFKs), as an inhibitor of MERS-CoV as well as other members of the Coronaviridae family. We evaluated the antiviral activity of saracatinib and found that it suppressed the early stages of the MERS-CoV life cycle in Huh-7 cells through a possible suppression of the SFK signaling pathways. Furthermore, we assessed the combined effect of saracatinib with another chemotherapeutic agent, gemcitabine, in Huh-7 cells. Enhanced effects with lower toxicity were observed in a combination of these two drugs. Data presented in this study suggest that saracatinib alone or in combination with other agents can provide a new therapeutic option for the treatment of MERS-CoV infection.

## 2. Materials and Methods

### 2.1. Cells and Viruses

African green monkey Vero (ATCC^®^ CCL-81) and human lung fibroblast MRC-5 (ATCC^®^ CCL-171) cells were purchased from American Tissue Culture Collection (ATCC^®^, Manassas, VA, USA). Human hepatoma Huh-7 and Crandell Reese feline kidney (CRFK) cells were obtained from Japan Cell Research Bank (National Institutes of Biomedical Innovation, Health and Nutrition, Japan) and Korean Cell Line Bank (KCBL, Seoul, Republic of Korea), respectively. Vero and Huh-7 cells were maintained in Dulbecco’s modified Eagle’s medium (DMEM, HyClone^TM^, Logan, UT, USA) supplemented with 10% fetal bovine serum (FBS, HyClone^TM^) at 37 °C in a 5% CO_2_ incubator. MRC-5 and CRFK cells were grown in minimal essential medium (MEM, HyClone^TM^) supplemented with 10% FBS at 37 °C in a 5% CO_2_ incubator.

Patient-derived isolate MERS-CoV (passage 4, MERS-CoV/KOR/KNIH/002_05_2015; GenBank accession No. KT029139.1) [18] was kindly provided by the Korea Centers for Disease Control and Prevention (KCDC, Osong, Republic of Korea). The working virus stock (passage 4) was propagated in Huh-7 cells. Briefly, the MERS-CoV/KOR/KNIH/002_05_2015 strain was inoculated into Huh-7 cells and incubated at 37 °C until >90% CPE was visible. The recombinant MERS-CoV (rMERS-CoV) and rMERS-CoV with mutation in spike 2 (S2) protein (designated rMERS-CoV S2) were passaged once on Vero cells upon receipt for stock preparation. The virus-containing supernatants were collected, clarified by centrifugation, and aliquots were stored at −80 °C until further use. Virus stocks were titrated by plaque assay using Vero cells as previously described [19]. All procedures using live MERS-CoV were performed in a biosafety level-3 facility at Virus Research Group, Korea Research Institute of Chemical Technology (KRICT), Daejeon, Republic of Korea. Human coronavirus 229E (HCoV-229E, ATCC^®^ VR-740) and betacoronavirus OC43 strain (OC43, ATCC^®^ VR-1558) were purchased from ATCC^®^ and amplified in MRC-5 cells. Feline infectious peritonitis virus (FIPV, ATCC^®^ VR-990) was purchased from ATCC and amplified in CRFK cells.

### 2.2. Chemical Compounds and Antibodies

Saracatinib (AZD0530; 99.9% purity) was purchased from Selleckchem (Houston, TX, USA) and gemcitabine hydrochloride (≥98% purity) was obtained from Sigma-Aldrich (St. Louis, MO, USA) for further analysis. All compounds were prepared in 100% dimethyl sulfoxide (DMSO, Sigma-Aldrich). Rabbit anti-MERS-CoV nucleocapsid (N) antibody was purchased from Sino Biological Inc. (Cat. 100211-RP02, Beijing, China). Antibodies against Fyn (4023), Lyn (2796), Src (2123), and Yes (3201) were purchased from Cell Signaling Technology (Danvers, MA, USA). The loading control, the anti-β-actin monoclonal antibody, was purchased from Sigma-Aldrich. Horseradish peroxidase (HRP)-conjugated goat anti-rabbit and anti-mouse secondary antibodies were purchased from Thermo Fisher Scientific (Waltham, MA, USA).

### 2.3. Small-Molecule Compound Libraries

The following compound libraries were used in this study: (i) the Pharmakon-1600 collection (Microsource Discovery Systems, Gaylordsville, CT, USA) comprised of FDA-approved drugs and drug substances approved for use in Europe or Asia; (ii) the Prestwick Chemical Library (Prestwick Chemical, San Diego, CA, USA) consisting of 326 mostly approved drugs (FDA, European Medicines Agency and other agencies); (iii) the Tocriscreen library (Tocris Bioscience, Bristol, United Kingdom) consisting of 103 FDA-approved compounds; and (iv) an in-house library comprised of 305 biologically active compounds including known drugs, experimental bioactive compounds, and pure natural products deposited in the Korea Chemical Bank at KRICT (Daejeon, Republic of Korea). All library compounds were dissolved in DMSO.

### 2.4. Primary Screening Assay

A CPE-inhibition assay was used to screen for inhibitors of MERS-CoV as previously described [19]. Briefly, Huh-7 cells were seeded in 96-well plates at a density of 2 × 10^4^ cells/well. After overnight incubation of the cells at 37 °C, equal volumes of MERS-CoV inoculum (multiplicity of infection [MOI] 0.1) and compound dilution (final concentration of 20 μM) were added and incubated at 37 °C in a 5% CO_2_ incubator for three days. On day three, post-infection (p.i.), cell viability was measured using the CellTiter 96^®^ AQ_ueous_ One Solution Cell Proliferation Assay (MTS, Promega, Madison, WI, USA), according to the manufacturer’s instructions. Absorbance at 490 nm (A490) was measured using a Synergy^TM^ H1 multi-mode microplate reader (Biotek, Winooski, VT, USA). Percent inhibition was calculated using the values of maximum infectivity derived from infected cultures treated with 0.5% DMSO (virus control, 0% inhibition) and background derived from mock-infected treated with 0.5% DMSO (cell control, 100% inhibition) as references using the formula ([sample − virus control]/[cell control − virus control] × 100). The statistical validity of the MERS-CoV primary screening system was determined by calculating for the Z’-factor using the values derived from virus control and cell control wells [20]. For the primary screening, a Z’-factor ≥0.5 and a coefficient of variation (CV) among the controls ≤10% was used to validate the results of the assay. The antiviral activity of hit compounds was determined from the dose-response curve (DRC), and the 50% effective concentration (EC_50_) and the compound-specific toxicity (50% cytotoxic concentration [CC_50_]) were calculated with GraphPad Prism 6 (GraphPad Software, La Jolla, CA, USA) using the non-linear regression formula: log (inhibitor) vs. response-variable response (four parameters) model.

### 2.5. Time-Of-Addition Assay

One day prior to infection, Huh-7 cells (5 × 10^4^ cells/well) were seeded in a 12-well tissue culture plate. The next day, cells were infected with MERS-CoV at an MOI 0.02 for 1 h at 37 °C. After 1 h, the unbound virus was removed by washing with phosphate-buffered saline (PBS, pH 7.4). Compounds were added to cells at specific time points during virus infection as follows: pre, 1 h prior to virus infection; co-treatment during virus infection; post, 1 h and 4 h after virus infection. The antiviral activity of the compound was analyzed after 24 h p.i. by measuring the number of infectious viral particles and intracellular viral RNA expression levels using plaque assay and RT-qPCR, respectively.

In addition, the time-of-addition experiment combined with temperature-shifting assay was performed. Briefly, Huh-7 cells were inoculated with MERS-CoV at 4 °C for 1 h (attachment/binding). The unadsorbed virus was removed by washing three times with ice-cold PBS and replenished with fresh culture medium. Subsequently, plates were shifted to 37 °C to allow synchronous entry and infection. Saracatinib was treated during the 4 °C incubation only or added at specific time points during the 37 °C incubation. The antiviral activity of the compound was analyzed after 24 h p.i. by measuring the number of infectious viral particles and intracellular viral RNA expression levels using plaque assay and RT-qPCR, respectively.

### 2.6. Viral Plaque Assay

Infectious MERS-CoV titers were determined by plaque assays as described previously (19). Briefly, Vero cells were seeded in 6-well plates at 5 × 10^5^ cells/well and cultured overnight at 37 °C in a 5% CO_2_ incubator. Cells were inoculated with ten-fold serially diluted cell culture supernatants for 1 h at 37 °C. After adsorption, cells were washed with PBS and overlaid with DMEM containing 0.5% agarose (MP Biomedicals, Solon, OH, USA) and 2% FBS. After three days of incubation, plaques were visualized by staining with 50 μg/mL neutral red (Sigma-Aldrich).

### 2.7. Quantitative Real-Time Reverse-Transcription PCR (RT-qPCR) Analysis

Total cellular RNA was purified using the RNeasy Mini kit (Qiagen, Valencia, CA, USA) according to the manufacturer’s instructions. The RT-qPCR was performed using a SuperScript III one-step RT-qPCR system with Platinum Taq polymerase (Thermo Fisher Scientific), primers/probe sets targeting *open reading frame 1a* (*ORF1a*) or *upE* genes (Table S1) and a QuantStudio 6 real-time PCR system (Applied Biosystems, Foster City, CA, USA) as described previously [21]. The relative viral RNA expression levels (fold change) were calculated by the ΔΔ*C_T_* method and glyceraldehyde-3-phosphate dehydrogenase (GAPDH) was used as the endogenous control. Two biological replicates, each with technical duplicates, were used for quantification.

### 2.8. Western Blot Analysis

Huh-7 cells were infected with MERS-CoV at an MOI of 0.02 or mock-infected and treated with ten-fold serial dilutions of each compound. At 24 h p.i., cells were washed with PBS and lysed using NP40 cell lysis buffer (Thermo Fisher Scientific) containing 0.5% protease inhibitor cocktail (Pierce, Rockford, IL, USA). The cell lysates were clarified by centrifugation and total protein content was determined by the Bradford Protein assay (Bio-Rad, Hercules, CA, USA). Equal amounts of protein were subjected to sodium dodecyl sulfate-polyacrylamide gel electrophoresis (SDS-PAGE) and electro-transferred to a polyvinylidene fluoride (PVDF) membrane (Immobilon^®^-P PVDF membrane, Merck Millipore, County Cork, Ireland). The membrane was blocked in Tris-buffered saline/T (0.1% Tween^®^20, Sigma-Aldrich) containing 5% skim milk for 2 h at room temperature. MERS-CoV N protein was detected using a primary antibody specific for viral N protein, followed by a horseradish peroxidase (HRP)-conjugated goat anti-rabbit secondary antibody. The cellular β-actin protein, a loading control, was detected with an anti-β-actin-specific primary antibody and HRP-conjugated goat anti-mouse secondary antibody. After the addition of a chemiluminescent HRP substrate (SuperSignal West Pico Chemiluminescent Substrate; Pierce), images were obtained using a LAS-4000 Luminescent Image Analyzer (Fujifilm, Tokyo, Japan).

### 2.9. RNAi

Huh-7 cells were transfected with pools of two or three specific small interfering RNAs (siRNAs) against human Fyn (GeneID 2534), Lyn (Gene ID 4067), Src (GeneID 6714) or Yes (GeneID 7525) or a siRNA universal negative control (Bioneer, Daejeon, Republic of Korea) to a final concentration of 100 nM by using Lipofectamine RNAiMAX transfection reagent (Thermo Fisher Scientific) according to the manufacturer’s instructions. Efficient knockdown of the targets was monitored by Western blot analysis at 48 h after siRNA transfection.

### 2.10. In Vitro Drug-Drug Combination Assay

To evaluate the drug combination effect in vitro, saracatinib was combined with gemcitabine. Different concentrations of a serially-diluted single compound were combined as dose matrices and MERS-CoV inhibition was evaluated by the CPE-inhibition assay as described above. Combination indices (CI) were calculated by using CompuSyn software (ComboSyn Inc., Paramus, NJ, USA) according to Loewes’ additivity model as described previously [22].

## 3. Results

### 3.1. Identification of Saracatinib as an Anti-MERS-CoV Hit Compound

**Figure 1:**
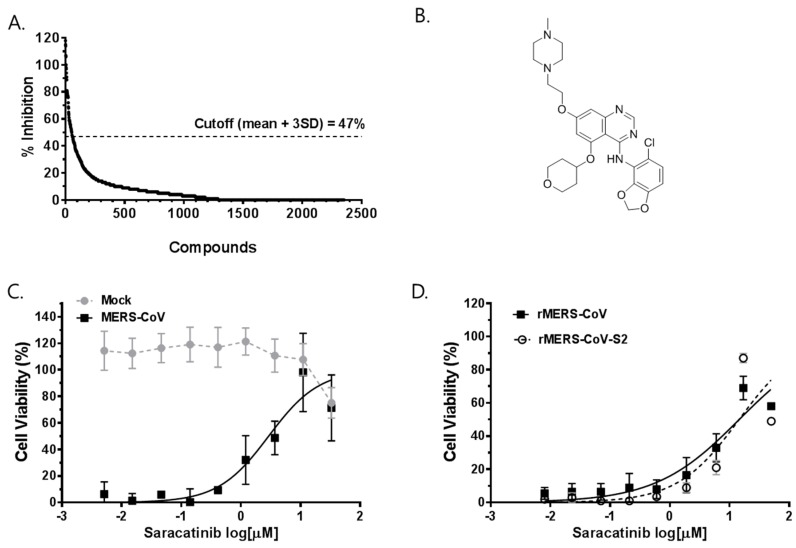
Identification of saracatinib as an anti-MERS-CoV inhibitor from screening of bioactive compound libraries. (**A**) The percentage inhibition of MERS-CoV induced-CPE from each compound in the primary screening. Each dot represents the individual compound tested. The hit identification was based on the calculation of average % inhibition ± 3 standard deviation (mean ± 3SD) cut-off, which in this study was 47% inhibition of the virus induced-CPE. (**B**) The structure of saracatinib. (**C**,**D**) Dose-response curve (DRC) analyses of the inhibition of MERS-CoV by saracatinib. Huh-7 cells were (**C**) mock-infected (grey circle) or infected with MERS-CoV (black square); (**D**) rMERS-CoV (black square) and rMERS-CoV-S2 (open circle) in the presence of various concentrations of saracatinib. At 72 h p.i., cell viability was measured using MTS-based CellTiter 96^®^ AQ_ueous_ One Solution Cell Proliferation Assay. The data represent means (±SD) of at least two independent experiments performed in duplicate.

In order to identify compounds with antiviral activity against MERS-CoV, we employed a cell-based antiviral screening assay based on the protection of cells from viral CPE. The primary screen of 2334 clinically approved drugs and pharmacologically active compounds (final concentration of 20 μM) identified 63 compounds with MERS-CoV antiviral activity above the cut-off (>47% inhibition), yielding a hit rate of 2.6% (Figure 1A). The average Z’ score during the primary screen was 0.80 ± 0.08, indicating that the assay was robust and reliable for the detection of potential MERS-CoV inhibitors (Figure S1). Further confirmation of the antiviral activity of selected compounds against MERS-CoV infection was performed by testing them in complete dose-response analyses ranging from 0.62 µM to 50 µM and the EC_50_ and CC_50_ values were determined. Using this method, we determined 12 hit compounds, primarily classified as antiprotozoal, anticancer and antipsychotics, active against MERS-CoV with micromolar EC_50_ values ranging from 2.1 µM to 14.4 µM (Table 1). Among the identified hits, saracatinib (AZD0530, Figure 1B) was particularly promising as it exhibited prominent antiviral activity with an estimated EC_50_ of 2.9 µM and a CC_50_ of >50 µM, resulting in selectivity index (SI) of approximately >17 (Figure 1C). Prior to proceeding further, we tested whether saracatinib displayed antiviral activity against different strains of MERS-CoV. Huh-7 cells were infected with the recombinant MERS-CoV (rMERS-CoV) derived from the EMC/2012 strain and a cell culture adapted variant derived from this virus (rMERS-CoV-S2). From the results, saracatinib exhibited an EC_50_ of 9.3 μM and 9.0 µM against rMERS-CoV and rMERS-CoV-S2, respectively, suggesting a broad-spectrum anti-MERS-CoV activity of the drug (Figure 1D). Moreover, saracatinib showed a broad-antiviral activity against other human coronaviruses such as hCoV-229E and OC43 with an EC_50_ 2.4 µM and 5.1 µM, respectively, and feline infectious peritonitis virus (FIPV) with an EC_50_ of 7 µM within a non-toxic range of concentrations (Table S2).

### 3.2. Saracatinib Inhibits MERS-CoV Replication In Vitro

**Figure 2:**
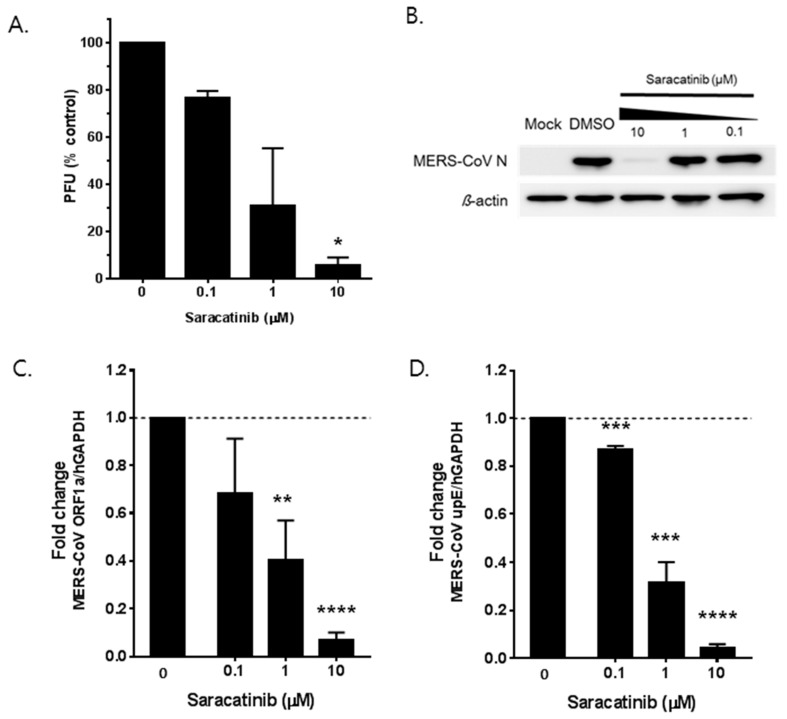
In vitro antiviral activity of saracatinib against MERS-CoV. Antiviral efficacy of saracatinib against MERS-CoV in Huh-7 cells. MERS-CoV infected Huh-7 cells were treated with saracatinib at indicated concentrations for 24 h, after which culture supernatant and cell lysates were collected. (**A**) Amount of infectious viral particles released to culture supernatants was determined by plaque assay. (**B**) MERS-CoV nucleocapsid (N) protein levels in lysates of infected cells were determined by Western blot analysis. Immunoblot detection of β-actin is shown as a loading control. (**C**,**D**) Quantification of intracellular MERS-CoV RNAs by RT-qPCR assay. Total RNA was isolated from lysates of infected cells for quantification of intracellular MERS-CoV RNA levels (*ORF1a* and *upE*) and results were normalized to GAPDH mRNA. Data represent means (±SD) of at least two independent experiments performed in duplicate. Significant differences are indicated by * *p* < 0.05, ** *p* < 0.01, *** *p* < 0.001, **** *p* < 0.0001.

To determine whether saracatinib directly inhibits MERS-CoV infection, we first assessed the effect of saracatinib on the production of MERS-CoV infectious progeny virus. The number of viral titers was measured in the cell culture supernatant of saracatinib-treated Huh-7 cells following the infection with MERS-CoV by the plaque assay. As shown in Figure 2A, a dose-dependent reduction in infectious MERS-CoV titer exceeding 50% was observed at 1 µM and close to 90% at 10 µM saracatinib as compared to untreated controls. Treatment of 0.1 µM saracatinib resulted in only marginal reduction of viral titer. Next, we assessed its impact on the expression of viral N protein and synthesis of MERS-CoV RNA by Western blot analysis and RT-qPCR, respectively. We infected Huh-7 cells with MERS-CoV at an MOI of 0.02 and harvested total cell lysate at 24 h p.i. Western blot analysis showed a nearly complete abrogation of viral N protein expression in infected cells treated with 10 µM saracatinib, whereas treatment of saracatinib at 1 µM or lower did not affect the expression of viral N protein levels (Figure 2B). Consistent with a reduction in viral titer, a dose-dependent reduction in both intracellular viral genomic (*ORF1a*; Figure 2C) and viral mRNAs (*upE*; Figure 2D) was observed in infected-cells treated with saracatinib when compared to those of untreated controls. Treatment of 10 µM saracatinib resulted in near complete inhibition of both intracellular viral genomic and mRNA syntheses. Together, these data confirm the in vitro anti-MERS-CoV activity of saracatinib by reducing the production of progeny virus as well as the expression of both viral RNA and protein levels at a micromolar range.

### 3.3. Saracatinib Inhibits the Early Stages of MERS-CoV Replication

**Figure 3:**
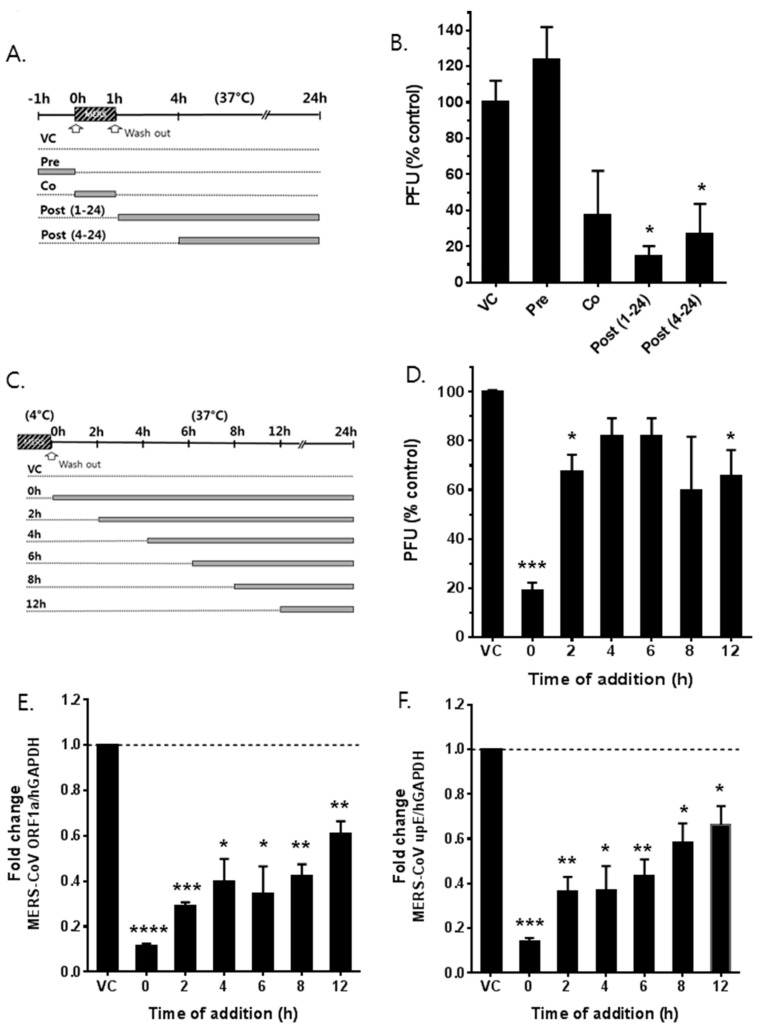
Saracatinib inhibits the early stages of MERS-CoV life cycle. (**A**) Schematic representation of time-of-addition/removal experiment. (**B**) Huh-7 cells were treated with 10 µM saracatinib for 1 h prior to virus infection (pre) or 0.5% DMSO (virus control, VC), for 1 h during infection (co), at 1 h post-infection (post 1–24), and at 4 h post-infection (post 4–24). After 24 h, the amount of infectious viral particles released to culture supernatants was determined by plaque assay. All values represent means ± SD of two independent experiments performed in duplicate. Significant differences are indicated by * *p* < 0.05. (**C**) Schematic representation of time-of-addition experiment. Huh-7 cells were inoculated with MERS-CoV at 4 °C to allow attachment/binding. After 1 h incubation, plates were shifted to 37 °C to allow synchronous entry and infection. Saracatinib (10 µM) was treated during the 4 °C incubation only or added at indicated time points during the 37 °C incubation and remained present until sample collection. After 24 h p.i.; (**D**) cell culture supernatants were collected for virus titration using plaque assay. (**E**,**F**) Total RNA isolated from infected lysates was used for analyses of intracellular MERS-CoV genomic RNA (*ORF1a*) and mRNA (*upE*) by qRT-qPCR. Data represent the means ± SD of two independent experiments performed in duplicate. Significant differences are indicated by * *p* < 0.05, ** *p* < 0.01, *** *p* < 0.001, **** *p* < 0.0001.

To investigate which step(s) of the MERS-CoV life cycle were affected by saracatinib, a time-of-addition/removal experiment was performed. Saracatinib (10 μM) was added to Huh-7 cells either 1 h prior to (pretreatment), during (co-treatment), 1 h or 4 h after infection (post-treatment) with MERS-CoV (Figure 3A). At 24 h p.i., the amount of released virus particles into the cell culture supernatant was determined by plaque assay. As shown in Figure 3B, saracatinib treatment prior to infection resulted in a similar viral titer when compared to untreated cells, indicating that the blockage of cellular receptor(s) was not its mode-of action. To clarify the possibility that saracatinib interacts directly with virus particles or cellular receptors, virus attachment was performed at 4 °C in the presence or absence of saracatinib. After 1 h adsorption, cells were washed and replenished with fresh media followed by incubation at 37 °C to allow virus internalization. The virus titer in cell culture supernatant from saracatinib-treated cells during incubation at 4 °C was similar to that of untreated cells, suggesting that saracatinib does not interact either with the virus particles or cellular receptors [23]. In contrast, the addition of the drug during virus infection at 37 °C resulted in a marked reduction of the viral titer (Figure 3B, co [*p* = 0.08]) as well as a significant decrease in the intracellular viral genomic RNA and mRNA expressions (Figure S2). Moreover, the reduction of MERS-CoV titer became more pronounced when 10 μM saracatinib was added 1 h after virus inoculation indicating that the drug interfered with the early stages of viral replication after internalization (Figure 3B, post [1,2,3,4,5,6,7,8,9,10,11,12,13,14,15,16,17,18,19,20,21,22,24,25]). The antiviral activity became less effective when saracatinib was added after 4 h post-inoculation (Figure 3B, post [4,5,6,7,8,9,10,11,12,13,14,15,16,17,18,19,20,21,22,24,25]). The inhibitory effect was prominent on the accumulation of intracellular MERS-CoV RNAs. The syntheses of both intracellular viral genomic RNA and mRNAs were severely affected upon treatment of the drug when added 1 h (post [1,2,3,4,5,6,7,8,9,10,11,12,13,14,15,16,17,18,19,20,21,22,24,25]) after virus inoculation (Figure S2). To more precisely understand the time window of the saracatinib-mediated inhibition of MERS-CoV replication, 10 μM saracatinib was added to infected cells at different time points after infection and remained present until sample collection (Figure 3C). At 24 h p.i., infectious particles and intracellular MERS-CoV genomic RNA and mRNAs were quantified as mentioned above. As shown in Figure 3D–F, saracatinib added within the first 2 h p.i. resulted in a significant reduction (~80%) in infectious virus titers and near complete inhibition of both viral genomic RNA and mRNA syntheses. Together, these data suggest that saracatinib most potently inhibits the early stages of MERS-CoV life cycle after internalization.

### 3.4. MERS-CoV Replication is Affected by Knockdown of Fyn and Lyn Kinases

**Figure 4:**
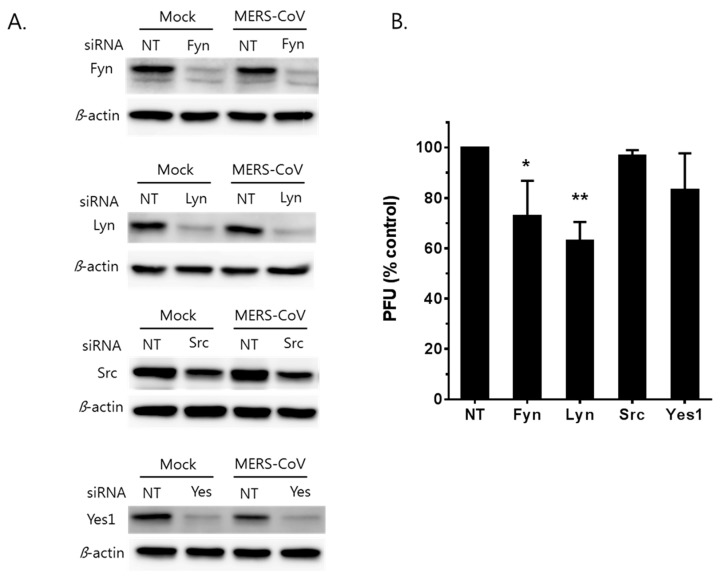
MERS-CoV is sensitive to RNA-mediated depletion of Fyn and Lyn kinases. (**A**) Specific knockdown of Fyn, Lyn, Src, and Yes kinases. Huh-7 cells were transfected with 100 nM nontargeting (NT) siRNA or siRNAs targeting Fyn, Lyn, Src, and Yes mRNA. At 48 h post-transfection, the cells were infected with MERS-CoV at an MOI of 0.02. After 12 h of infection, lysates of infected cells were collected and subjected to Western blot analyses. The β-actin was used as the loading control. The result of a representative experiment out of two repeats is shown. (**B**) Specific knockdowns of Fyn and Lyn interfere with the production of infectious MERS-CoV from siRNA transfected cells. Data presented is from means ± SD of 3 independent experiments. Significant differences are indicated by * *p* < 0.05, ** *p* < 0.01.

As previously mentioned, saracatinib has significant inhibitory activity against several members of SFKs as well as Abelson (Abl) kinase [24,25]. Next, we investigated the role(s) of members of SFKs (Fyn, Lyn, Src, and Yes) in the MERS-CoV replication expressed in Huh-7 cells. Using a RNAi-based approach, expression of these kinases was knocked down in Huh-7 cells prior to MERS-CoV infection. Significant knockdown of targeted kinases was achieved in Huh-7 cells as confirmed by Western blot analyses at 48 h post-siRNA transfection (Figure 4A). The siRNA-transfected cells were infected with MERS-CoV at an MOI of 0.02. At 12 h p.i., we observed significant reductions in virus titer when Huh-7 cells were knocked down of Fyn and Lyn kinases with siRNA (Figure 4B). In contrast, no significant inhibition of MERS-CoV replication was observed in Yes-knocked down cells. The extent of Src kinase knock down was not efficient enough to determine the role of the kinase during MERS-CoV replication in our study. Nevertheless, the data suggest that expression of Fyn and Lyn kinases may be necessary for efficient MERS-CoV replication.

### 3.5. Synergistic Antiviral Effect of Saracatinib in Combination with Gemcitabine

**Figure 5:**
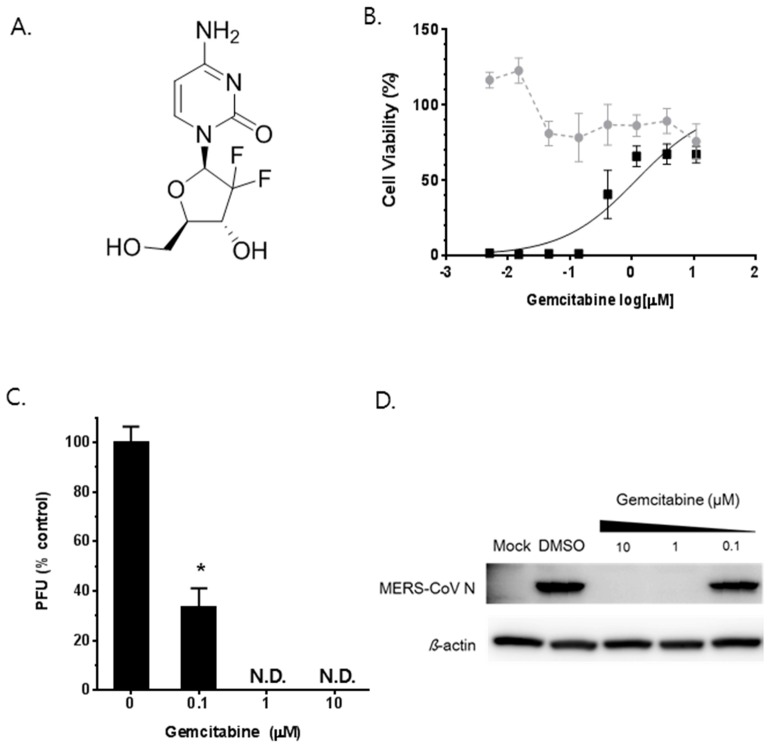
In vitro antiviral activity of gemcitabine against MERS-CoV. (**A**) The structure of gemcitabine. (**B**) Antiviral potency of gemcitabine against MERS-CoV. The antiviral EC_50_ (black squares) and CC_50_ (grey circles) of gemcitabine were determined by dose-response curve (DRC) analyses as described in Figure 2. (**C**,**D**) Antiviral efficacy of gemcitabine against MERS-CoV in Huh-7 cells. MERS-CoV infected Huh-7 cells were treated with gemcitabine at indicated concentrations for 24 h, after which the culture supernatant and cell lysates were collected. (**C**) Amount of infectious viral particles released to culture supernatants was determined by plaque assay. (**D**) MERS-CoV N protein levels in lysates of infected cells were determined by Western blot analysis. The immunoblot detection of β-actin is shown as a loading control. Data represent means (±SD) of at least two independent experiments performed in duplicate. Significant differences are indicated by * *p* < 0.05.

Finally, we explored the possibility of using saracatinib as a combinatorial therapy with other clinically available drugs. Gemcitabine is a deoxycytidine analog that is commonly used for the treatment of cancers [26,27] (Figure 5A). Recently, several studies have shown its broad-spectrum antiviral effect on enteroviruses and highly pathogenic coronaviruses including MERS-CoV and SARS-CoV [14,28]. Prior to analyzing the efficacy of 2-drug combination, we confirmed the anti-MERS-CoV potential of gemcitabine in our cell-based assays. Briefly, gemcitabine treatment showed a convincing dose-dependent antiviral effect in Huh-7 cells with an EC_50_ of 1.2 µM at a non-cytotoxic concentration range (Figure 5B). Subsequently, treatment with a 1 µM or higher dose of gemcitabine completely abolished the production of infectious MERS-CoV particles as well as the expression of viral N protein expression assessed by plaque assay and Western blot analysis, respectively (Figure 5C,D).

**Figure 6:**
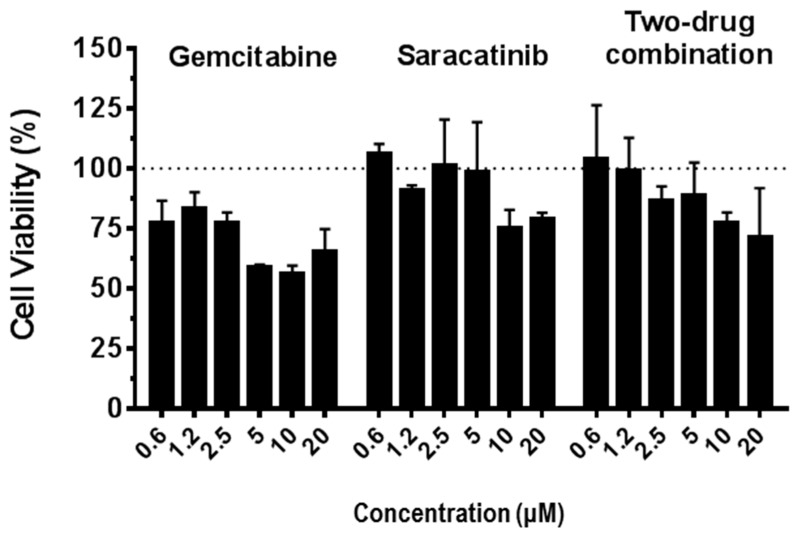
Cytotoxicity of gemcitabine and saracatinib, alone or in combination, in Huh-7 cells. Cells were treated with increasing concentrations of gemcitabine or saracatinib alone, or in combination for 48 h and cell viability was measured using the CellTiter 96^®^ AQ_ueous_ One Solution Cell Proliferation Assay. Data represent means (±SD) of at least two independent experiments performed in duplicate.

Next, we investigated the effects of saracatinib in combination with gemcitabine. MERS-CoV infected Huh-7 cells were simultaneously treated with a dose range of 0, 1/8, 1/4, 1/2, 1, 2, and 4 of EC_50_ of saracatinib and gemcitabine and the combined antiviral effect was assessed using the colorimetric cell viability assay (Figure S3). The combination index (CI) was calculated according to the methods of Chou et al. [14] and analyzed using CompuSyn software. The 2-drug combination of saracatinib with gemcitabine showed a considerable synergistic antiviral effect with a CI value of 0.529 (CI < 1, =1, and >1 indicated synergism, additive effect, and antagonism, respectively; Table 2). Moreover, it is noteworthy that the combined treatment with 2-drugs showed no differential cytotoxicity when compared to that of saracatinib alone. Importantly, a combination of saracatinib with gemcitabine showed less cytotoxicity than gemcitabine alone in Huh-7 cells, indicating that it is suitable for use in low-dose combination therapy than the individual drugs used alone (Figure 6). Together, these results suggest that saracatinib can be used effectively in combination with gemcitabine against MERS-CoV infection.

## 4. Discussion

In this study, we described the antiviral activity of saracatinib against MERS-CoV infection in Huh-7 cells. Saracatinib (AZD0530), developed for the treatment of tumor malignancies [25], was identified by screening anti-MERS-CoV activity of 2334 approved drugs and biologically active molecules using a CPE-based HTS assay. From the primary screening, we identified several drugs including chloroquine, amodiaquine, and chloropromazine analogs that have previously been reported as inhibitors of the MERS-CoV by other groups [13,14], indicating the validity of our screening results. In addition, we found two neurotransmitter inhibitors, dosulepin and hydroxyzine pamoate, with different chemical core structures to those previously reported that inhibited the replication of MERS-CoV in vitro at micromolar concentrations. Moreover, a protein kinase C inhibitor, sotrastaurin, undergoing clinical trials for the prevention of organ transplant rejection and psoriasis treatment [29], also blocked MERS-CoV infection in Huh-7 cells with an EC_50_ of approximately 9.7 μM with low cellular toxicity (CC_50_ > 50 μM). However, the SI of these molecules were limited (<10) when compared to that of saracatinib, which was selected for further studies.

Saracatinib is an orally available small molecule that are potent SFK members and Abl kinases by blocking their ATP-binding sites [25]. The SFKs and related kinases play a central role in the regulation of multiple signal transduction pathways involved in gene transcription, cytoskeleton organization, and cell proliferation that are essential for viral replication [30,31,32]. Several research groups have demonstrated that SFKs are associated with diverse aspects of viral infections including the induction of the antiviral immune response [33,34] as well as the facilitation of viral entry such as coxsackievirus, influenza A virus, Ebola virus, and Kaposi’s sarcoma-associated herpesvirus [35,36,37,38,39], RNA replication of hepatitis C virus [40,41], West Nile virus assembly [42], and regulation of multiple stages of human immunodeficiency virus-1 replication [43,44,45,46].

Our data showed that saracatinib inhibited MERS-CoV replication by reducing virus titers at a micromolar range. The production of infectious virus particles as well as syntheses of viral genomic RNA and mRNAs were severely affected when saracatinib was added to cells and left for the first 4 h of virus infection while its antiviral effect was significantly reduced when the drug was added after 4 h of infection, suggesting that the drug inhibits the early stages of the MERS-CoV life cycle. Subsequently, we aimed to identify the target(s) of saracatinib involved in MERS-CoV infection by the RNAi-depletion of Fyn, Lyn, Src, and Yes, expressed in Huh-7 cells. Our data showed that knockdown of Fyn or Lyn resulted in a significant reduction in MERS-CoV titers, although the effects were less severe when compared to those of saracatinib treatment. The difference could be explained by the fact that saracatinib is a pan-SFK selective agent that potently inhibits several members of SFKs as well as other nonreceptor tyrosine kinases such as Abl [25], suggesting that multiple kinases might be involved in MERS-CoV infection. In addition, the ubiquitous expression of some members of SFKs leads to a high level of functional redundancy where the remaining SFK members compensate for those that are functionally inactive. Regrettably, we could not definitely determine whether the depletion of Src kinase activity alone or in combination with other members of SFKs had a detrimental effect on MERS-CoV replication due to the experimental limitations of using siRNA-based knockdown assays. This is particularly important since previous studies have demonstrated that multiple members of SFKs, especially the Src, Fyn, and Yes kinases, often in conjunction with Abl kinase, play an important role in the life cycle of various viruses [39,47,48,49]. Recently, Coleman et al. indicated that Abl2, the imatinib target, was required for efficient SARS-CoV and MERS-CoV replication in vitro [17]. In a follow up study, Sisk et al. [50] demonstrated that Abl kinase activity and associated pathways were involved in the coronavirus fusion step with endosomal membrane as well as cell-cell fusion that occurs late in infection. Thus, we speculate that the anti-MERS-CoV activity of saracatinib is mediated similarly via the inhibition of multiple members of SFKs including Fyn/Lyn together with Abl2 kinase. The exact mechanism of the action of multiple members of SFKs together with Abl2 kinase and associated pathways involved in MERS-CoV replication is currently under investigation in our laboratory.

Due to the central function of individual SFKs in many signaling pathways such as the EGF receptor (EGFR), Ras/Raf/MEK, PI3K/AKT, and JAK/STAT pathways [30,51] involved in cell metabolism, there are other possible mechanisms by which Fyn/Lyn or other members of SFKs may affect the infectious cycle of MERS-CoV and other coronaviruses. Along these lines, a previous study on the kinome analysis of MERS-CoV infection indicated that ERK/MAPK intermediates, in particular MEK1/2 and ERK1/2, play important roles during the course of infection [16]. Similarly, in the closely related murine coronavirus mouse hepatitis virus (MHV), inhibitory effects on phosphorylation of the substrate ERK1/2 by specific inhibitor of MEK1/2 resulted in the strong suppression of MHV propagation at the step of viral RNA synthesis in culture cells [52,53]. From these findings, we inferred that the suppression of SFK resulted in the negative regulation of downstream signaling responses such as ERK/MAPK and PI3K/AKT/mTOR pathways, which in turn have adverse effects on coronavirus replication. However, the effects of saracatinib on these various signaling pathways remain to be further elucidated.

Several FDA-approved tyrosine kinase inhibitors are actively used in the clinic or in clinical trials for cancer treatment, however, increasing evidence has shown that cells acquire resistance to these agents by activating a modified signaling pathway to replace the lack of a signal in target therapy [54]. To overcome these limitations, we evaluated the combined effects of saracatinib with another chemotherapeutic agent, gemcitabine, with a different mechanism of action to compensate for any drug resistance that may arise. Gemcitabine, an FDA-approved anticancer drug, is a deoxycytidine analog that interferes with normal DNA/RNA synthesis through the inhibition of ribonucleoside reductase [55]. Previous studies have shown that gemcitabine exhibits a broad spectrum antiviral effect against several RNA viruses including the influenza virus, enteroviruses, and coronaviruses [14,28,56,57,58]. Co-treatment of saracatinib with gemcitabine showed a synergistic antiviral effect and minimal cytotoxic effect, supporting the idea of using them in a combination therapy to potentiate efficacy at non-toxic concentrations as well as to overcome any drug resistance issues.

In summary, this study suggested that saracatinib shows antiviral activity at the early stages of the MERS-CoV life cycle after internalization into Huh-7 cells through a possible mechanism of suppressing SFK signaling pathways. In addition, we identified Fyn and Lyn kinases as additional host factors involved in MERS-CoV infection. Thus, our results support the view that host kinases, such as SFKs, play important role in coronavirus infection. Similarly, other FDA-approved kinase inhibitors can be tested for antiviral activity against human coronaviruses and used as tools to identify novel host kinases involved in virus replication. Moreover, the in vitro synergistic antiviral effect of saracatinib in combination with gemcitabine against MERS-CoV infection suggests that lower therapeutic dosage may be used to prevent any toxic side effect that the drug may have at therapeutic concentration. Taken together, these findings provide insight into the potential use of clinically validated SFK kinase inhibitors for the future development of new therapeutic options for highly pathogenic coronavirus infection such as MERS-CoV.

## Figures and Tables

**Table 1:** Hit compounds with antiviral activity against MERS-CoV.

No.	Compound	EC_50_ (µM) ^†^	CC_50_ (μM) ^†^	Pharmaceutical Class	Mode of Action
1	Nutlin-3	6.9 ± 1.4	26.8 ± 1.6	Antiviral, anticancer	MDM2 inhibitor
2	Hydroxyzine pamoate	14.4 ± 3.4	>50	Antihistamine	Cholinergic system modulator
3	Amodiaquine dihydrochloride ^§^	2.1 ± 0.7	12.3 ± 5.9	Antiprotozoal	Histamine N-methyltransferase inhibitor
4	Amodiaquine dihydrochloride dehydrate ^§^	2.4 ± 0.7	13.2 ± 5.0	Antiprotozoal	Histamine N-methyltransferase inhibitor
5	Chloroquine diphosphate ^§^	12.0 ± 3.0	>50	Antiprotozoal	Hemozoin formation inhibitor
6	Hydroxychloroquine sulfate ^§^	13.3 ± 2.1	>50	Antiprotozoal	Hemozoin formation inhibitor
7	Saracatinib	2.9 ± 0.6	57 ± 5.5	Anticancer	Tyrosine kinase inhibitor
8	Sotrastaurin	9.7 ± 3.3	>50	Anticancer	Protein kinase C inhibitor
9	Acetophenazine maleate	11.2 ± 5.0	23.6 ± 3.8	Antipsychotic	Dopamine receptor antagonist
10	Dosulepin hydrochloride	3.4 ± 0.0	28.9 ± 0.0	Antidepressant	Serotonin/noradrenaline reuptake inhibitor
11	Methotrimeprazine maleate salt	2.5 ± 0.0	24.5 ± 0.0	Antipsychotic	Dopamine receptor antagonist
12	N1-(4-pyridyl)-2-chloro-5-nitrobenzamide	10.5 ± 0.3	>50		

^§^ MERS-CoV antiviral activity also described by Dyall et al. [14]. ^†^ The 50% effective concentration (EC_50_) and the 50% cytotoxic concentration (CC_50_) were calculated using the non-linear regression formula.

**Table 2:** Synergistic antiviral effect of saracatinib and gemcitabine.

Drug Combination	Combination Ratio	^a^ CI Values at Inhibition of				^b^ Weighted Average CI Values	^c^ Graded Symbols	Description
		50%	75%	90%	95%			
Saracatinib + Gemcitabine	1:1	1.260	0.696	0.434	0.334	0.529	+++	Synergism

^a^ Data analysis was performed using CompuSyn software (ComboSyn, Inc.). Data represent mean of the average of two independent experiments (*n* = 2). ^b^ Weighted average combination index (CI) values were determined as CI_wt_ = [CI_50_ + 2 × CI_75_ + 3 × CI_90_ + 4 × CI_95_]/10. ^c^ Degree of synergism (+ signs) is based on the ranges of CI values as described previously [22].

## Supplementary Materials

The following are available online at http://www.mdpi.com/1999-4915/10/6/283/s1, Table S1. Primer and probe sequences used in the RT-qPCR assay. Table S2. Antiviral activity of saracatinib against other members of the Coronaviridae family. Figure S1. Assay validation of chemical library screening. Each dot represents the Z’ score calculated from the cell control and virus control in each plate. Brifely, the Z’ score was calculated using the formula: 1 − [(3*σρ* + 3*ση*)(|*μρ* − *μη*|)], where the *μρ*, *σρ*, and *ση* represent the means (*μ*) and standard deviations (*σ*) of the positive (*ρ*) and negative (*η*) controls [20]. Figure S2. Saracatinib inhibits the early stages of the MERS-CoV life cycle. Huh-7 cells were treated with 10 µM saracatinib for 1 h prior to virus infection (pre) or 0.5% DMSO (virus control, VC), for 1 h during infection (co), at 1 h post-infection (post 1–24), and at 4 h post-infection (post 4–24). After 24 h p.i., the total RNA isolated from the infected lysates were collected and used for analyses of intracellular MERS-CoV genomic RNA (*ORF1a*, left panel) and mRNA (*upE*, right panel) by RT-qPCR. Data represent means ± SD of two independent experiments performed in duplicate. Significant differences are indicated by * *p* < 0.05, ** *p* < 0.01, *** *p* <0.001, **** *p* < 0.0001. Figure S3. Synergistic antiviral effect of saracatinib and gemcitabine. MERS-CoV infected Huh-7 cells were simultaneously treated with a dose range of 1/2, 1, 2, and 4 of EC_50_ of saracatinib and gemcitabine and the combined antiviral effect was assessed using the CellTiter 96^®^ AQ_ueous_ One Solution Cell Proliferation Assay. Data represent means ± SD of two independent experiments performed in duplicate.

## References

[1] De Wit E., van Doremalen N., Falzarano D., Munster V.J. (2016). SARS and MERS: Recent insights into emerging coronaviruses. Nat. Rev. Microbiol..

[2] Zaki A.M., van Boheemen S., Bestebroer T.M., Osterhaus A.D., Fouchier R.A. (2012). Isolation of a novel coronavirus from a man with pneumonia in Saudi Arabia. N. Engl. J. Med..

[3] Zumla A., Chan J.F., Azhar E.I., Hui D.S., Yuen K.Y. (2016). Coronaviruses—Drug discovery and therapeutic options. Nat. Rev. Drug Discov..

[4] Chan J.F., Li K.S., To K.K., Cheng V.C., Chen H., Yuen K.Y. (2012). Is the discovery of the novel human betacoronavirus 2c EMC/2012 (HCoV-EMC) the beginning of another SARS-like pandemic?. J. Infect..

[5] Chan J.F., Lau S.K., Woo P.C. (2013). The emerging novel Middle East respiratory syndrome coronavirus: The “knowns” and “unknowns”. J. Formos. Med. Assoc..

[6] Azhar E.I., El-Kafrawy S.A., Farraj S.A., Hassan A.M., Al-Saeed M.S., Hashem A.M., Madani T.A. (2014). Evidence for camel-to-human transmission of MERS coronavirus. N. Engl. J. Med..

[7] Drosten C., Meyer B., Muller M.A., Corman V.M., Al-Masri M., Hossain R., Madani H., Sieberg A., Bosch B.J., Lattwein E. (2014). Transmission of MERS-coronavirus in household contacts. N. Engl. J. Med..

[8] Oboho I.K., Tomczyk S.M., Al-Asmari A.M., Banjar A.A., Al-Mugti H., Aloraini M.S., Alkhaldi K.Z., Almohammadi E.L., Alraddadi B.M., Gerber S.I. (2015). 2014 MERS-CoV outbreak in Jeddah—A link to health care facilities. N. Engl. J. Med..

[9] Zumla A., Hui D.S., Perlman S. (2015). Middle East respiratory syndrome. Lancet.

[10] Chan J.F., Lau S.K., To K.K., Cheng V.C., Woo P.C., Yuen K.Y. (2015). Middle East respiratory syndrome coronavirus: Another zoonotic betacoronavirus causing SARS-like disease. Clin. Microbiol. Rev..

[11] Al-Tawfiq J.A., Memish Z.A. (2014). What are our pharmacotherapeutic options for MERS-CoV?. Expert Rev. Clin. Pharmacol..

[12] Ashburn T.T., Thor K.B. (2004). Drug repositioning: Identifying and developing new uses for existing drugs. Nat. Rev. Drug Discov..

[13] De Wilde A.H., Jochmans D., Posthuma C.C., Zevenhoven-Dobbe J.C., van Nieuwkoop S., Bestebroer T.M., van den Hoogen B.G., Neyts J., Snijder E.J. (2014). Screening of an FDA-approved compound library identifies four small-molecule inhibitors of Middle East respiratory syndrome coronavirus replication in cell culture. Antimicrob. Agents Chemother..

[14] Dyall J., Coleman C.M., Hart B.J., Venkataraman T., Holbrook M.R., Kindrachuk J., Johnson R.F., Olinger G.G., Jahrling P.B., Laidlaw M. (2014). Repurposing of clinically developed drugs for treatment of Middle East respiratory syndrome coronavirus infection. Antimicrob. Agents Chemother..

[15] Hart B.J., Dyall J., Postnikova E., Zhou H., Kindrachuk J., Johnson R.F., Olinger G.G., Frieman M.B., Holbrook M.R., Jahrling P.B. (2014). Interferon-beta and mycophenolic acid are potent inhibitors of Middle East respiratory syndrome coronavirus in cell-based assays. J. Gen. Virol..

[16] Kindrachuk J., Ork B., Hart B.J., Mazur S., Holbrook M.R., Frieman M.B., Traynor D., Johnson R.F., Dyall J., Kuhn J.H. (2015). Antiviral potential of ERK/MAPK and PI3K/AKT/mTOR signaling modulation for Middle East respiratory syndrome coronavirus infection as identified by temporal kinome analysis. Antimicrob. Agents Chemother..

[17] Coleman C.M., Sisk J.M., Mingo R.M., Nelson E.A., White J.M., Frieman M.B. (2016). Abelson Kinase Inhibitors Are Potent Inhibitors of Severe Acute Respiratory Syndrome Coronavirus and Middle East Respiratory Syndrome Coronavirus Fusion. J. Virol..

[18] Kim D.W., Kim Y.J., Park S.H., Yun M.R., Yang J.S., Kang H.J., Han Y.W., Lee H.S., Kim H.M., Kim H. (2016). Variations in Spike Glycoprotein Gene of MERS-CoV, South Korea, 2015. Emerg. Infect. Dis..

[19] Kumar V., Shin J.S., Shie J.J., Ku K.B., Kim C., Go Y.Y., Huang K.F., Kim M., Liang P.H. (2017). Identification and evaluation of potent Middle East respiratory syndrome coronavirus (MERS-CoV) 3CLPro inhibitors. Antivir. Res..

[20] Zhang J.H., Chung T.D., Oldenburg K.R. (1999). A Simple Statistical Parameter for Use in Evaluation and Validation of High Throughput Screening Assays. J. Biomol. Screen..

[21] Go Y.Y., Kim Y.S., Cheon S., Nam S., Ku K.B., Kim M., Cho N.H., Park H., Alison Lee P.Y., Lin Y.C. (2017). Evaluation and Clinical Validation of Two Field-Deployable Reverse Transcription-Insulated Isothermal PCR Assays for the Detection of the Middle East Respiratory Syndrome-Coronavirus. J. Mol. Diagn..

[22] Chou T.C. (2006). Theoretical basis, experimental design, and computerized simulation of synergism and antagonism in drug combination studies. Pharmacol. Rev..

[23] Go Y.Y. (2018). Evaluation of Antiviral Activity of Saracatinib against Virus Attachment.

[24] Green T.P., Fennell M., Whittaker R., Curwen J., Jacobs V., Allen J., Logie A., Hargreaves J., Hickinson D.M., Wilkinson R.W. (2009). Preclinical anticancer activity of the potent, oral Src inhibitor AZD0530. Mol. Oncol..

[25] Hennequin L.F., Allen J., Breed J., Curwen J., Fennell M., Green T.P., Lambert-van der Brempt C., Morgentin R., Norman R.A., Olivier A. (2006). *N*-(5-chloro-1,3-benzodioxol-4-yl)-7-[2-(4-methylpiperazin-1-yl)ethoxy]-5-(tetrahydro-2*H*-pyran-4-yloxy)quinazolin-4-amine, a novel, highly selective, orally available, dual-specific c-Src/Abl kinase inhibitor. J. Med. Chem..

[26] Plunkett W., Huang P., Gandhi V. (1995). Preclinical characteristics of gemcitabine. Anticancer Drugs.

[27] Plunkett W., Huang P., Xu Y.Z., Heinemann V., Grunewald R., Gandhi V. (1995). Gemcitabine: Metabolism, mechanisms of action, and self-potentiation. Semin. Oncol..

[28] Kang H., Kim C., Kim D.E., Song J.H., Choi M., Choi K., Kang M., Lee K., Kim H.S., Shin J.S. (2015). Synergistic antiviral activity of gemcitabine and ribavirin against enteroviruses. Antivir. Res..

[29] Manicassamy S. (2009). Sotrastaurin, a protein kinase C inhibitor for the prevention of transplant rejection and treatment of psoriasis. Curr. Opin. Investig. Drugs.

[30] Thomas S.M., Brugge J.S. (1997). Cellular functions regulated by Src family kinases. Annu. Rev. Cell Dev. Biol..

[31] Ricono J.M., Huang M., Barnes L.A., Lau S.K., Weis S.M., Schlaepfer D.D., Hanks S.K., Cheresh D.A. (2009). Specific cross-talk between epidermal growth factor receptor and integrin αvβ5 promotes carcinoma cell invasion and metastasis. Cancer Res..

[32] Colicelli J. (2010). ABL tyrosine kinases: Evolution of function, regulation, and specificity. Sci. Signal.

[33] Johnsen I.B., Nguyen T.T., Bergstroem B., Fitzgerald K.A., Anthonsen M.W. (2009). The tyrosine kinase c-Src enhances RIG-I (retinoic acid-inducible gene I)-elicited antiviral signaling. J. Biol. Chem..

[34] Johnsen I.B., Nguyen T.T., Ringdal M., Tryggestad A.M., Bakke O., Lien E., Espevik T., Anthonsen M.W. (2006). Toll-like receptor 3 associates with c-Src tyrosine kinase on endosomes to initiate antiviral signaling. EMBO J..

[35] Hahn A.S., Kaufmann J.K., Wies E., Naschberger E., Panteleev-Ivlev J., Schmidt K., Holzer A., Schmidt M., Chen J., Konig S. (2012). The ephrin receptor tyrosine kinase A2 is a cellular receptor for Kaposi’s sarcoma-associated herpesvirus. Nat. Med..

[36] Eierhoff T., Hrincius E.R., Rescher U., Ludwig S., Ehrhardt C. (2010). The epidermal growth factor receptor (EGFR) promotes uptake of influenza A viruses (IAV) into host cells. PLoS Pathog..

[37] Kumar N., Sharma N.R., Ly H., Parslow T.G., Liang Y. (2011). Receptor tyrosine kinase inhibitors that block replication of influenza a and other viruses. Antimicrob. Agents Chemother..

[38] Brindley M.A., Hunt C.L., Kondratowicz A.S., Bowman J., Sinn P.L., McCray P.B., Quinn K., Weller M.L., Chiorini J.A., Maury W. (2011). Tyrosine kinase receptor Axl enhances entry of Zaire ebolavirus without direct interactions with the viral glycoprotein. Virology.

[39] Coyne C.B., Bergelson J.M. (2006). Virus-induced Abl and Fyn kinase signals permit coxsackievirus entry through epithelial tight junctions. Cell.

[40] Pfannkuche A., Buther K., Karthe J., Poenisch M., Bartenschlager R., Trilling M., Hengel H., Willbold D., Haussinger D., Bode J.G. (2011). c-Src is required for complex formation between the hepatitis C virus-encoded proteins NS5A and NS5B: A prerequisite for replication. Hepatology.

[41] Supekova L., Supek F., Lee J., Chen S., Gray N., Pezacki J.P., Schlapbach A., Schultz P.G. (2008). Identification of human kinases involved in hepatitis C virus replication by small interference RNA library screening. J. Biol. Chem..

[42] Hirsch A.J., Medigeshi G.R., Meyers H.L., DeFilippis V., Fruh K., Briese T., Lipkin W.I., Nelson J.A. (2005). The Src family kinase c-Yes is required for maturation of West Nile virus particles. J. Virol..

[43] Tintori C., Laurenzana I., La Rocca F., Falchi F., Carraro F., Ruiz A., Este J.A., Kissova M., Crespan E., Maga G. (2013). Identification of Hck inhibitors as hits for the development of antileukemia and anti-HIV agents. ChemMedChem.

[44] McCarthy S.D., Sakac D., Neschadim A., Branch D.R. (2016). c-SRC protein tyrosine kinase regulates early HIV-1 infection post-entry. AIDS.

[45] Musumeci F., Schenone S., Brullo C., Desogus A., Botta L., Tintori C. (2015). Hck inhibitors as potential therapeutic agents in cancer and HIV infection. Curr. Med. Chem..

[46] Coiras M., Ambrosioni J., Cervantes F., Miro J.M., Alcami J. (2017). Tyrosine kinase inhibitors: Potential use and safety considerations in HIV-1 infection. Expert Opin. Drug Saf..

[47] De Wispelaere M., LaCroix A.J., Yang P.L. (2013). The small molecules AZD0530 and dasatinib inhibit dengue virus RNA replication via Fyn kinase. J. Virol..

[48] Kumar R., Agrawal T., Khan N.A., Nakayama Y., Medigeshi G.R. (2016). Identification and characterization of the role of c-terminal Src kinase in dengue virus replication. Sci. Rep..

[49] Newsome T.P., Weisswange I., Frischknecht F., Way M. (2006). Abl collaborates with Src family kinases to stimulate actin-based motility of vaccinia virus. Cell. Microbiol..

[50] Sisk J.M., Frieman M.B., Machamer C.E. (2018). Coronavirus S protein-induced fusion is blocked prior to hemifusion by Abl kinase inhibitors. J. Gen. Virol..

[51] Finn R.S. (2008). Targeting Src in breast cancer. Ann. Oncol..

[52] Cai Y., Liu Y., Zhang X. (2007). Suppression of coronavirus replication by inhibition of the MEK signaling pathway. J. Virol..

[53] Cai Y., Liu Y., Zhang X. (2006). Induction of transcription factor Egr-1 gene expression in astrocytoma cells by Murine coronavirus infection. Virology.

[54] Sierra J.R., Cepero V., Giordano S. (2010). Molecular mechanisms of acquired resistance to tyrosine kinase targeted therapy. Mol. Cancer.

[55] Mini E., Nobili S., Caciagli B., Landini I., Mazzei T. (2006). Cellular pharmacology of gemcitabine. Ann. Oncol..

[56] Denisova O.V., Kakkola L., Feng L., Stenman J., Nagaraj A., Lampe J., Yadav B., Aittokallio T., Kaukinen P., Ahola T. (2012). Obatoclax, saliphenylhalamide, and gemcitabine inhibit influenza a virus infection. J. Biol. Chem..

[57] Clouser C.L., Patterson S.E., Mansky L.M. (2010). Exploiting drug repositioning for discovery of a novel HIV combination therapy. J. Virol..

[58] Song J.H., Kim S.R., Heo E.Y., Lee J.Y., Kim D.E., Cho S., Chang S.Y., Yoon B.I., Seong J., Ko H.J. (2017). Antiviral activity of gemcitabine against human rhinovirus in vitro and in vivo. Antivir. Res..

