# Supplementary material for: Saracatinib Inhibits Middle East Respiratory Syndrome-Coronavirus Replication In Vitro
Source: Viruses. 2018 May 24;10(6):283. doi: 10.3390/v10060283 (PMC6024778; doi:10.3390/v10060283)

Table S1. Primer and probe sequences used in RT-qPCR assay.

Target	Primers/Probe	Sequence (5′-3′)
ORF1a	Forward	CCACTACTCCCATTTCGTCAG
	Reverse	CAGTATGTGTAGTGCGCATATAAGCA
	Probe	FAM-TTGCAAATTGGCTTGCCCCCACT-TAMRA
UpE	Forward	GCAACGCGCGATTCAGTT
	Reverse	GCCTCTACACGGGACCCATA
	Probe	FAM-CTCTTCACATAATCGCCCCGAGCTCG-BHQ1
hGAPDH	Forward	GAAGGTGAAGGTCGGAGTC
	Reverse	GAAGATGGTGATGGGATTTC
	Probe	HEX-CAAGCTTCCCGTTCTCAGCC-BHQ1

Table S2. Antiviral activity of saracatinib against other members of *Coronaviridae* family.

	**Saracatinib**	
	**CC_{50} (μM)**	**EC_{50} (μM)**
HCoV-229E	11	2.4
HCoV-OC43	11	5.1
Feline infectious peritonitis virus (FIPV)	>50	7

Figure S1

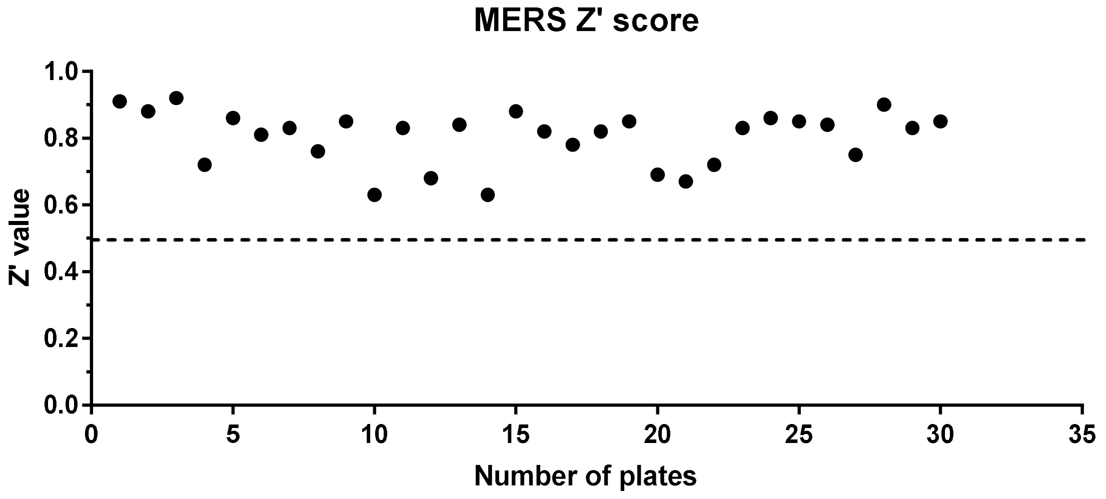

Figure S2

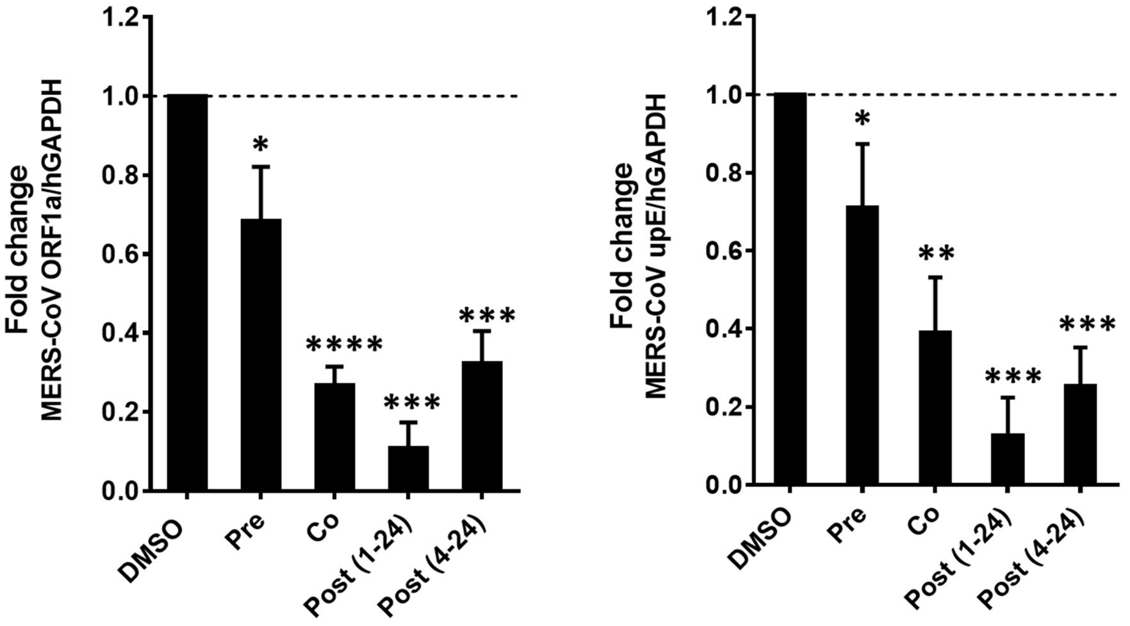

Figure S3

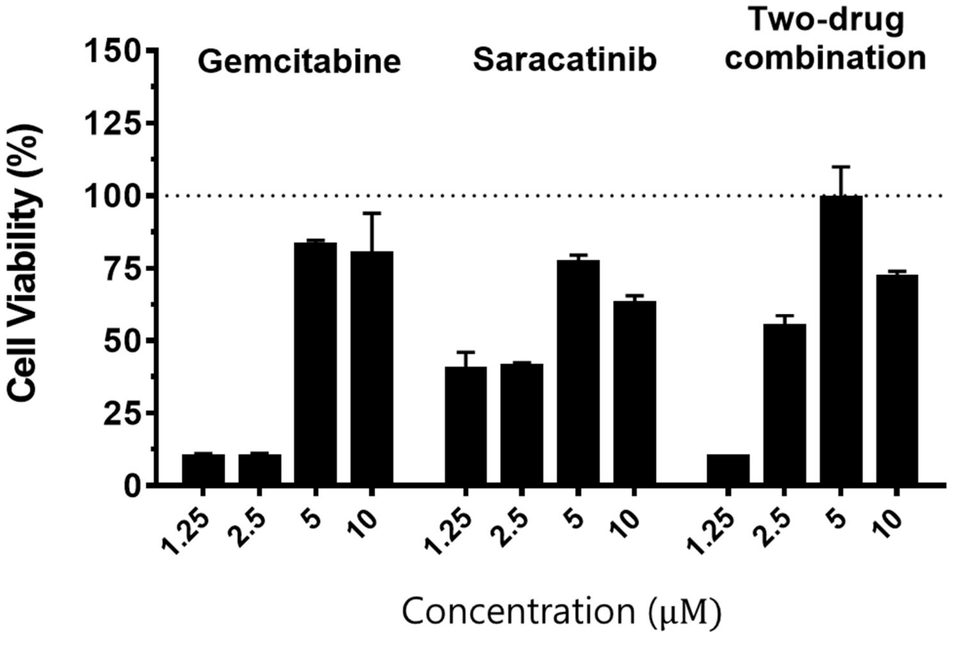

Supplement: Supplementary file 1 [file viruses-10-00283-s001.pdf]
